# Investigating the Effect of Lignosulfonate on Erosion Rate of the Embankments Constructed with Clayey Sand

**DOI:** 10.1155/2013/587462

**Published:** 2013-12-29

**Authors:** Hamid Reza Koohpeyma, Amir Hossein Vakili, Hossein Moayedi, Alireza Panjsetooni, Ramli Nazir

**Affiliations:** ^1^Department of Civil Engineering, Estahban Branch, Islamic Azad University, Estahban 74518-64747, Iran; ^2^School of Civil Engineering, Universiti Sains Malaysia, 14300 Nibong Tebal, Penang, Malaysia; ^3^Faculty of Engineering, Kermanshah University of Technology, Kermanshah, Iran; ^4^Department of Geotechnics and Transportation, Faculty of Civil Engineering, Universiti Teknologi Malaysia, 81310 Skudai, Johor, Malaysia

## Abstract

Internal erosion is known as the most important cause of dam failure after overtopping. It is important to improve the erosion resistance of the erodible soil by selecting an effective technique along with the reasonable costs. To prevent internal erosion of embankment dams the use of chemical stabilizers that reduce the soil erodibility potential is highly recommended. In the present study, a lignin-based chemical, known as lignosulfonate, is used to improve the erodibility of clayey sand specimen. The clayey sand was tested in various hydraulic heads in terms of internal erosion in its natural state as well as when it is mixed with the different percentages of lignosulfonate. The results show that erodibility of collected clayey sand is very high and is dramatically reduced by adding lignosulfonate. Adding 3% of lignosulfonate to clayey sand can reduce the coefficient of soil erosion from 0.01020 to 0.000017. It is also found that the qualitative erodibility of stabilized soil with 3% lignosulfonate is altered from the group of extremely rapid to the group of moderately slow.

## 1. Introduction

Due to the large volume of water behind the dam, destroying dams causes enormous financial and human tragedies. Internal erosion of the earth dams is considered the second cause of dam failure after overtopping from the dam crest [1]. It is the process that increases the concentrated leakage and can spread cavities leading to the destruction of the dam with an uncontrollable and catastrophic drainage of reservoir [2]. To examine the process of erosion and piping, the pinhole test was devised for identifying erodible soils [3]. The Teton dam is one example of this problem that was caused by internal erosion. The dam was demolished in 1976 and caused a number of deaths in Southeastern Idaho, and the property damage was about 1.0 billion USD [4]. Thus, studying and understanding this phenomenon and its factors is very important.

One of the effective methods to prevent the internal erosion is the use of stabilizers to reduce the erodibility of soils [5]. In recent years, chemical additives such as cement, lime, and fly ash have been widely used in the construction projects to stabilize various erodible soils [6–10]. However, conventional agents such as cement, lime, and fly ash due to occupational health or safety outcomes are not environmentally friendly. They will also increase the alkalinity of ground water. The stabilized soil by traditional additives has normally a pH higher than 9, which often affects the durability of reinforcement of concrete and steel frames of buildings [5]. In addition, other chemical characteristics such as electrical conductivity and ion exchange capacity of the soil are reduced as the amount of additives and processing time decreases and this, in turn, affects the water holding capacity [11–14].

In order to overcome these outcomes, it is required to use another soil stabilizer that can improve the strength and durability of the soil which does not harm the environment [15]. Recently, a lignin-based chemical, lignosulfonate, has shown promising aspects in the stabilization of problematic soil [5, 16]. Lignosulfonate is a brown substance with a pH of about 4, is flammable, and based on National Occupational Health And Safety Commission criteria is not considered a hazardous material. Previous researchers have also shown that the clay particles of treated soil by lignosulfonate could be successfully aggregated. This is because the neutralization process takes place and the lignosulfonate with positive charge is attracted by negative charges of clay minerals [15, 16]. Accordingly, the thickness of the diffuse double layer of particles affected by lignosulfonate is significantly decreased [15, 17].

Assessment of the lignosulfonate potential to improve the soil with rapid erosion rate was the main aim of the current study. Therefore, the erodibility of clayey sand and the effect of lignosulfonate on it were experimentally investigated in this study.

## 2. Material and Methods

### 2.1. The Soil Used in This Study

**Figure 1:**
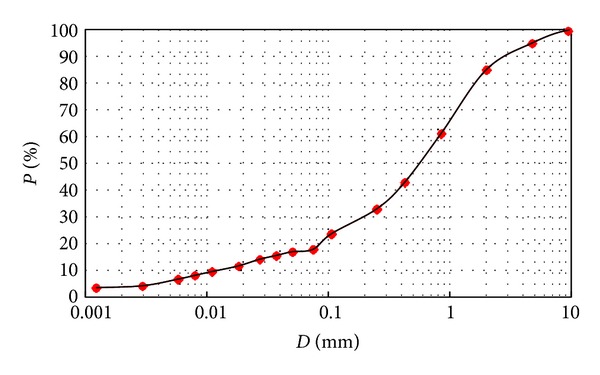
The gradation curve of the clayey sand.

The soil used in this investigation was clayey sand soil, collected from Marand area located in East Azerbaijan province in Iran. Initially the collected soil was passed through the sieve No. 10, thereafter, to classify the soil; the particle size distribution and Atterberg limit tests were done based on the ASTM D 422 and ASTM D 4318 standards, respectively. The gradation curve and properties of the used soil are observed in Figure 1 and Table 1, respectively. In addition, the maximum dry density of collected soil and its optimum moisture content were determined based on ASTM D 698 standard and were found to be 1.880 (g/cm^3^) and 11.5%, respectively.

### 2.2. Preparing the Sample

**Figure 2:**
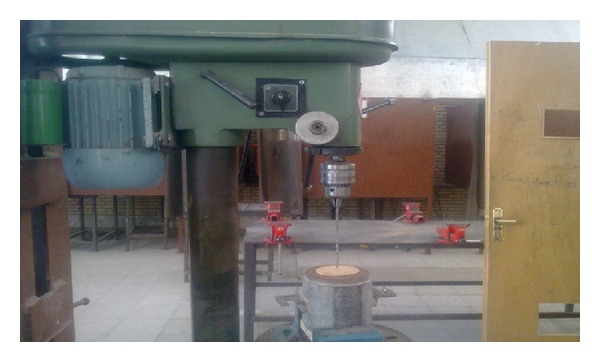
A view of performing a hole with drill along the longitudinal axis of compacted ample.

To prepare the stabilized samples, various percentages of lignosulfonate including 0.4, 1, 2, and 3% by dry mass of soils were initially mixed with water. The required amount of water was calculated from compaction test to obtain the optimum moisture content and maximum dry density. Subsequently, the mixture of water-lignosulfonate was added uniformly to the soil specimens. Then, the samples were kept in the sealed plastic containers and cured for 7 days to complete the stabilization process due to addition of lignosulfonate to soil. Next, the stabilized soil was compacted within the mold of hole erosion test device with a hammer of the standard compaction. Thereafter, the two sides of the mold were covered with plastic, and the sample was kept within the mold for three more hours; this is done to reach equilibrium between the particles after being compaction. As observed in Figure 2, preparing the sample was completed by drilling a 6 mm hole in diameter through its longitudinal axis. In reality, the concentrated leak within the embankment is simulated by performing the mentioned hole [1].

When the sample is prepared, the mold is placed in the Hole Erosion Test device. After sealing, the downstream valve is slowly opened to set the downstream water head. In this stage, it is important to fill the upper and lower containers accurately since the lower section of the sample starts to be destroyed if the containers are filled quickly, and this can lead to blockage of the hole. It should be mentioned that tap water was used to carry out the hole erosion test.

### 2.3. Internal Erosion Test

The test employed in this study is a specialized test for internal erosion called Hole Erosion Test (HET). The described details by Wan and Fell [1, 18] were followed to fabricate the HET equipment [1, 18]. The fabricated HET is capable of applying water head up to 1850 mm by which it is possible to evaluate the erosion rate of soils with low erodibility. This test could be performed to measure the rate of erosion and critical hydraulic shear stress [1]. The minimum hydraulic shear stress which is required to initiate the erosion is defined as a critical hydraulic shear stress [5]. The diameter of the used sample in this test is more than that of other tests that can simulate internal erosion. This test is employed because of its simplicity, low cost, and reliability of its results compared with other tests [19].

After performing Hole Erosion Tests on the prepared samples and analyzing data, the graphs of the erosion rate against time, hydraulic shear stress versus time, and the hydraulic shear stress versus erosion rate were drawn based one (1) and (2):

(1) $$\begin{array}{c} \tau_{t}=\rho_{w}gS_{t}\frac{\phi_{t}}{4}, \end{array}$$

in which *τ*_*t*_ refers to the hydraulic shear stress on the surface of the created hole at time *t*  (N/m^2^); *ρ*_*w*_ is density of the eroding fluid; *g* is the acceleration of gravity (9.8 m/s^2^); *S*_*t*_ refers to hydraulic gradient in soil samples at the time *t*; and *ϕ*_*t*_ is diameter of the pre-formed hole in time *t* (m). It should be added that the diameter of the hole is calculated using the indirect measurement method by determining flow rate during testing:

(2) $$\begin{array}{c} {\dot{\varepsilon }}_{t}=\frac{\rho_{d}}{2}\frac{d\phi_{t}}{dt}, \end{array}$$

where ${\dot{\varepsilon }}_{t}$ is the rate of erosion and *ρ*_*d*_ is the dry density of the soil (kg/m^3^).

The unstabilized and stabilized samples were categorized in terms of erodibility according to Table 3 which has been proposed by Wan and Fell [1] and is known as qualitative classification. In this table, the *I* is the erosion rate index which is calculated according to the following formula:

(3) $$\begin{array}{c} I=-\log C_{e}, \end{array}$$

where *C*_*e*_ is the coefficient of soil erosion which is obtained from the hydraulic shear stress graph versus erosion rate of the soil. In other words, *C*_*e*_ is defined as a slope of best fitted line in the ascending part of the mentioned graph. According to Table 2, there are six groups to classify soils in terms of erodibility. Noticeably, the greater *I* value indicate the less erodibility of the soil.

## 3. Result and Discussions

Initially, the effects of lignosulfonate on compaction characteristics of the clayey sand soil were determined. Table 3 gives the compaction test results of clayey sand and its compositions with lignosulfonate in different percentages of 0.4, 1, 2, and 3 based on the ASTM D 698 standard.

**Figure 3:**
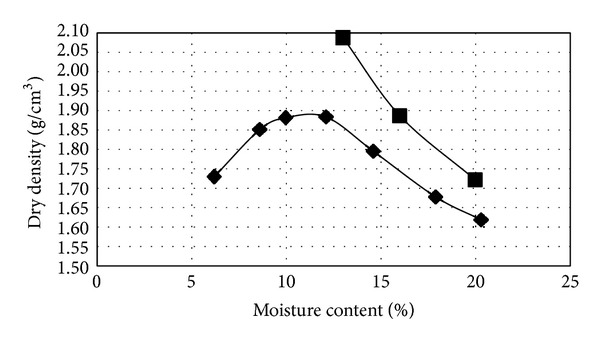
Compaction graph of clayey sand.

**Figure 4:**
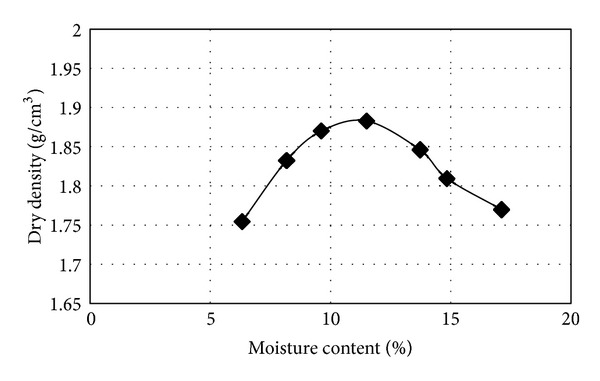
Compaction graph of stabilized clayey sand with 3% lignosulfonate.

It can be concluded that lignosulfonate will not affect the compaction characteristics including maximum dry density and optimum moisture content, since lignosulfonate is completely soluble in water and the amount of use is minimal. This can be evidenced by comparing the compaction graph of clayey sand (Figure 3) and the compaction graph of clayey sand stabilized with 3% lignosulfonate (Figure 4). It is worthy to note that this trend has been also reported by previous researchers [16].

**Figure 5:**
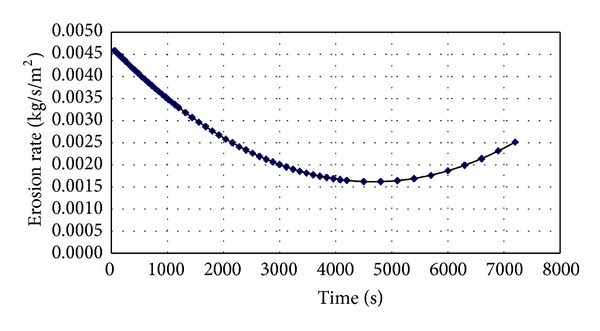
The variation of erosion rate versus time.

The variation of erosion rate versus time for a stabilized sample is presented in Figure 5. On the other hand, the trend of soil erosion within the mold and the disturbing impact of the sample during the drilling action is also reflected in Figure 5.

**Figure 6:**
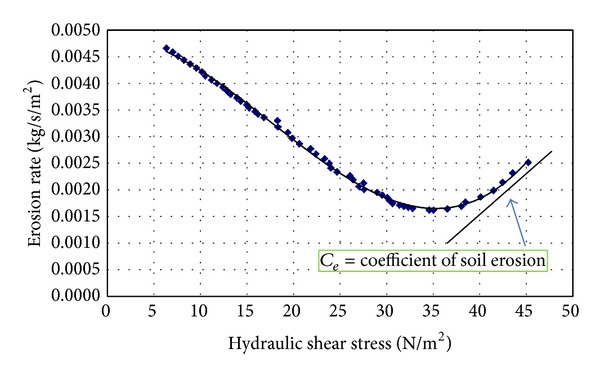
The erosion rate versus hydraulic shear stress.

The erosion rate against hydraulic shear stress of a soil sample is presented in Figure 6. The erosion rate index varies for different samples. The nearly-linear part of the graph shows erosion in the sample. The initial part of the graph shows a leaching and disposal of the disturbed soil inside the hole. This is due to drilling action and it cannot indicate the inherent erosion of the sample.

Table 4 shows the erosion rate index obtained from the internal erosion experiments on the clayey sand for both unstabilized and stabilized specimens. As stated earlier the stabilization process was performed with different percentages of lignosulfonate where the head varied (i.e., 50, 100, 200, 300, and 400 mm). In addition, the qualitative classification of the specimens in terms of erodibility is given in Table 5. As observed in Table 5, the soil sample stabilized by 3 percent lignosulfonate is classified into group of moderately slow, whereas, unstabilized sample is known as soil with erosion rate of extremely rapid.

As it can be seen in Tables 4 and 5, as the percentage of lignosulfonate is increased, the erodibility of the materials is significantly decreased (increase of erosion rate index). In fact, the effectiveness of lignosulfonate in stabilization process could be observed better in higher hydraulic gradients. In reality, there is a rapid flow rate in higher gradients; however, lignosulfonate prevents the penetration of flow into soil particles by creating a polymer chain between the particles and leading the soil particles neither to be dislodged and to move rapid. It is worth noting that in high hydraulic gradients, sandy soil samples containing clay which have not been stabilized with lignosulfonate have a high intention of erodibility. In the head of 100 mm and higher, the unstabilized soil samples were completely torn away and the soil was removed from the wall of the test mold that indicates rapid erosion of the sample. Lignosulfonate in high gradients showed more resistance and has a remarkable performance, so in the head of 400 mm by adding only 3% of the chemical stabilizer to the soil, the qualitative erodibility was changed from the extremely rapid group to the moderately slow group, and the coefficient of soil erosion was reduced from 0.01020 to 0.000017.

**Figure 7:**
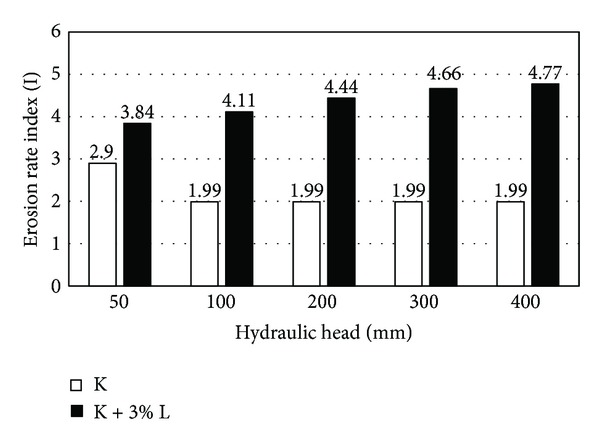
Comparison of the erosion rate index for the unstabilized and stabilized samples.

The erosion rate index of clayey sand stabilized with 3% lignosulfonate and unstabilized clayey sand in different hydraulic heads is compared in Figure 7. It can be concluded that the lignosulfonate has a significant influence in amendment of erodible soil which was used in this study.

**Figure 8:**
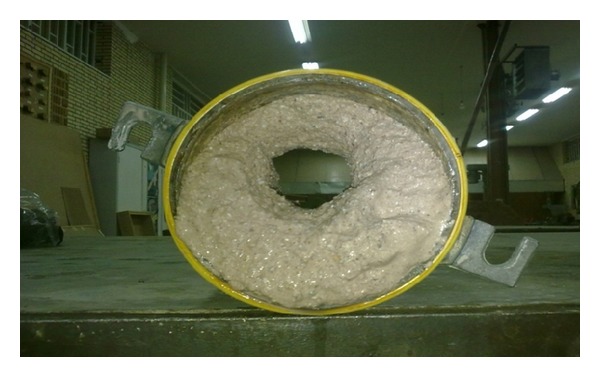
The hole size of unstabilized soil after performing the hole erosion test.

**Figure 9:**
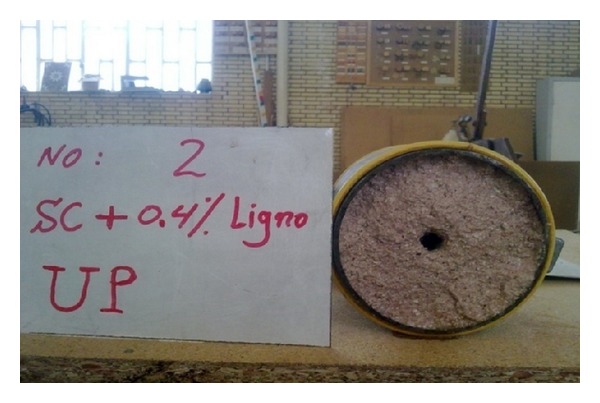
The hole size of stabilized soil by 0.4% lignosulfonate after performing the Hole Erosion Test.

After combining with soil, lignosulfonate creates a polymer chain between soil particles to prevent erosion. On the other hand, the soil aggregates are formed due to stabilization process by lignosulfonate [15, 17]. The function of lignosulfonate in reducing the erodibility of clayey sand could be observed by comparing both Figures 8 and 9.  Figure 8 shows the image of the final diameter of the pre-formed hole for simulation of the concentrated leakage and initiation of erosion on clayey sand after conducting the hole erosion test.  Figure 9 shows the final diameter of the pre-formed hole in the clayey sand stabilized with 0.4% lignosulfonate after the hole erosion test. A significant reduction was recorded in the final hole size of stabilized sample by only 0.4% lignosulfonate. Consequently, the erosion rate of high erodible soils like clayey sand which was obtained from Marand district could be substantially reduced by mixing the soli with low concentration of lignosulfonate content.

## 4. Conclusion

In this study, the effect of stabilization with lignosulfonate on reducing the erodibility of clayey sand was examined using the hole erosion test. The following results are drown from this study.

- The soil under study shows a severe internal erosion and, in fact, can be classified as the soil with extremely rapid erosion rate.
- Adding 0.4% lignosulfonate can reduce the soil erosion rate to a great extent.
- Adding 3% lignosulfonate to clayey sand will reduce the coefficient of soil erosion from 0.01020 to 0.000017 and will cause qualitative erodibility to change from the group of extremely rapid to the group of moderately slow.
- Lignosulfonate shows a more effective performance in the higher hydraulic gradients.
- The compaction characteristics of clayey sand specimen including its maximum dry density and its optimum moisture content were not strongly affected by mixing it with lignosulfonate.

## Figures and Tables

**Table 1:** The classification of the soil used.

LL (%)	PL (%)	PI (%)	Soil classification	Type of soil
25.35	14.22	11.13	SC	Clayey sand

Note: LL: liquid limit; PL: plastic limit; PI: plasticity index.

**Table 2:** Qualitative classification of soil in terms of erodibility [18].

Group number	Erosion rate index (*I*)	Description
1	<2	Extremely rapid
2	2-3	Very rapid
3	3-4	Moderately rapid
4	4-5	Moderately slow
5	5-6	Very slow
6	>6	Extremely slow

**Table 3:** Compaction characteristics of unstabilized and stabilized samples by different percentages of lignosulfonate.

Soil type	Maximum dry density (g/cm^3^)	Optimum moisture content (%)
K	1.880	11.55
K + 0.4% L	1.875	11.79
K + 1% L	1.870	11.75
K + 2% L	1.872	11.79
K + 3% L	1.870	11.60

**Table 4:** Erosion rate index for unstabilized and stabilized samples by lignosulfonate in various hydraulic heads.

Soil combination	*H* = 50 mm	*H* = 100 mm	*H* = 200 mm	*H* = 300 mm	*H* = 400 mm
K	2.90	<2	<2	<2	<2
K + 0.4% L	3.52	3.80	4.11	4.17	4.32
K + 1% L	3.63	3.90	4.14	4.22	4.38
K + 2% L	3.68	3.95	4.25	4.28	4.64
K + 3% L	3.84	4.11	4.44	4.66	4.77

Note: K: clayey sand, L: lignosulfonate.

**Table 5:** Qualitative classification of unstabilized and stabilized samples by lignosulfonate in various hydraulic heads based on hole erosion test results.

Soil combination	*H* = 50 mm	*H* = 100 mm	*H* = 200 mm	*H* = 300 mm	*H* = 400 mm
K	Very rapid	Extremely rapid	Extremely rapid	Extremely rapid	Extremely rapid
K + 0.4% L	Moderately rapid	Moderately rapid	Moderately slow	Moderately slow	Moderately slow
K + 1% L	Moderately rapid	Moderately rapid	Moderately slow	Moderately slow	Moderately slow
K + 2% L	Moderately rapid	Moderately rapid	Moderately slow	Moderately slow	Moderately slow
K + 3% L	Moderately rapid	Moderately slow	Moderately slow	Moderately slow	Moderately slow

Note: K: clayey sand, L: lignosulfonate.
